# Melanoma-inhibiting activity (MIA) mRNA is not exclusively transcribed in melanoma cells: low levels of MIA mRNA are present in various cell types and in peripheral blood

**DOI:** 10.1038/sj.bjc.6690808

**Published:** 1999-11

**Authors:** T J de Vries, A Fourkour, C J A Punt, H Diepstra, D J Ruiter, G N P van Muijen

**Affiliations:** Departments of Pathology andMedical Oncology, University Hospital Nijmegen, PO Box 9101, Nijmegen HB 6500, The Netherlands

**Keywords:** MIA, melanoma, circulating cancer cells, RT-PCR

## Abstract

The detection of minimal amounts of melanoma cells by tyrosinase reverse transcription polymerase chain reaction (RT-PCR) is seriously hampered by false negative reports in blood of melanoma patients with disseminated melanoma. Therefore, additional assays which make use of multiple melanoma markers are needed. It has been shown that introduction of multiple markers increases the sensitivity of detection. Melanoma inhibitory activity (MIA) is one such melanoma-specific candidate gene. To test the specificity of MIA PCR, we performed 30 and 60 cycles of PCR with two different sets of MIA specific primers on 19 melanoma and 16 non-melanoma cell lines. MIA mRNA was detected in 16 out of 19 melanoma cell lines and in seven out of 16 non-melanoma cell lines after 30 cycles of PCR. However, MIA mRNA could be detected in all cell lines after 60 cycles of PCR. Also, in 14 out of 14 blood samples of melanoma patients, five out of six blood samples of non-melanoma patients and in seven out of seven blood samples of healthy volunteers, MIA mRNA was detected after 60 cycles of PCR, whereas no MIA PCR product could be detected in any of the blood samples after 30 cycles of PCR. We conclude that low levels of MIA transcripts are present in various normal and neoplastic cell types. Therefore, MIA is not a suitable marker gene to facilitate the detection of minimal amounts of melanoma cells in blood or in target organs of the metastatic process. © 1999 Cancer Research Campaign


*British Journal of Cancer* (1999) **81**(6), 1066–1070
© 1999 Cancer Research Campaign
Article no. bjoc.1999.0808

### Melanoma-inhibiting activity (MIA) mRNA is not exclusively transcribed in melanoma cells: low levels of MIA mRNA are present in various cell types and in peripheral blood

**TJ de Vries[1]*, A Fourkour[1], CJA Punt[2], H Diepstra[1], DJ Ruiter[1] and GNP van Muijen[1]**

Departments of [1]Pathology and [2]Medical Oncology, University Hospital Nijmegen, PO Box 9101, 6500 HB Nijmegen, The Netherlands

**Summary** The detection of minimal amounts of melanoma cells by tyrosinase reverse transcription polymerase chain reaction (RT-PCR) is seriously hampered by false negative reports in blood of melanoma patients with disseminated melanoma. Therefore, additional assays which make use of multiple melanoma markers are needed. It has been shown that introduction of multiple markers increases the sensitivity of detection. Melanoma inhibitory activity (MIA) is one such melanoma-specific candidate gene. To test the specificity of MIA PCR, we performed 30 and 60 cycles of PCR with two different sets of MIA specific primers on 19 melanoma and 16 non-melanoma cell lines. MIA mRNA was detected in 16 out of 19 melanoma cell lines and in seven out of 16 non-melanoma cell lines after 30 cycles of PCR. However, MIA mRNA could be detected in all cell lines after 60 cycles of PCR. Also, in 14 out of 14 blood samples of melanoma patients, five out of six blood samples of non-melanoma patients and in seven out of seven blood samples of healthy volunteers, MIA mRNA was detected after 60 cycles of PCR, whereas no MIA PCR product could be detected in any of the blood samples after 30 cycles of PCR. We conclude that low levels of MIA transcripts are present in various normal and neoplastic cell types. Therefore, MIA is not a suitable marker gene to facilitate the detection of minimal amounts of melanoma cells in blood or in target organs of the metastatic process. © 1999 Cancer Research Campaign

**Keywords:** MIA; melanoma; circulating cancer cells; RT-PCR

The introduction in 1991 of a tyrosinase-specific reverse transcription polymerase chain reaction (RT-PCR) which can sensitively detect occult melanoma cells in peripheral blood (Smith et al, 1991), has created perspectives to carefully monitor dissemination of melanoma cells. The clinical relevance of this detection method has been demonstrated by various groups. Shorter survival is observed in patients with a positive tyrosinase PCR outcome (Kunter et al, 1996; Mellado et al, 1996) and semi-quantitative tyrosinase PCR results correlate with response to treatment (Brossart et al, 1995).

Although it was originally reported that tyrosinase mRNA could be detected in all patients with distant metastases (Brossart et al, 1993), various groups could not reproduce this high sensitivity of detection (reviewed in Keilholz et al, 1997). The use of multiple melanoma-specific markers increases the sensitivity of detection (Hoon et al, 1995; Curry, 1998). We recently described that a nested PCR for the melanoma antigen MART-1 (*m*elanoma *a*n*t*igen *r*ecognized by *T*-cells) is more sensitive than the widely-used tyrosinase PCR (De Vries et al, 1999), although others have previously described that tyrosinase PCR is more sensitive than MART-1 PCR (Curry et al, 1998). In our study, both tyrosinase and MART-1 PCR were extremely specific: no amplification was found in non-melanoma blood samples even after 90 cycles of PCR (60 first PCR, and 30 nested PCR) (De Vries et al, 1999). Other melanoma antigens such as gp100, Muc-18 and p97 were less specific, since the mRNAs of these antigens were detected by PCR in blood of normal volunteers (Brouwenstijn et al, 1997; Curry et al, 1998). Similarly, Burchill and co-workers found expression of cytokeratins 8 and 19 in peripheral blood (Burchill et al, 1995), excluding these cytokeratins as markers for circulating carcinoma cells.

In the present study, we tested the value of a specific RT-PCR for melanoma inhibitory activity (MIA) in the detection of melanoma cells in peripheral blood. MIA has been described as a gene which was widely expressed in melanoma cell lines and not expressed in peripheral blood, as investigated by Northern blotting (Blesch et al, 1994). Interestingly, glioma cells also expressed MIA, raising the possibility that MIA is more generally expressed in neuroectodermal tissues (Blesch et al, 1994). Also, after 25 cycles of RT-PCR, MIA could not be detected in a wide variety of non-melanoma cell lines, but it was detected at low levels in skin biopsies and at high levels in primary melanoma and melanoma metastasis lesions (Bosserhoff et al, 1996).

This reported melanocyte- and/or neuroectodermal specificity of MIA and the parallel with tyrosinase (Wimmer et al, 1997) that the MIA protein is present in serum of melanoma patients at increased levels with disease (Bosserhoff et al, 1997), prompted us to investigate whether a MIA-specific RT-PCR can be used for the detection of melanoma cells in the circulation. A pilot study promisingly showed that MIA PCR is suitable and more sensitive

*Received 26 January 1999*
*Revised 13 April 1999*
*Accepted 22 April 1999*

*Correspondence to:* GNP van Muijen

*Present address: Department of Cell Biology and Histology, Academic Medical Center, University of Amsterdam, PO Box 22700, 1100 DE Amsterdam, The Netherlands

**Table 1** Primer sequences.

| Target | Primers | Sequence[a] | s/as[b] | Product size |
|---|---|---|---|---|
| PBGD | PBGD-5′ | 5′-CTGGTAACGGCAATGCGGCT-3′ | s | 339 bp |
| | PBGD-3′ | 5′-GCAGATGGCTCCGATGGTGA-3′ | a | |
| MIA | MIA1 | 5′-CCAAGTGGTGTATGTCTTCTCCAA-3′ | s | 191 bp |
| | MIA2 | 5′-GGCAGTAGAAATCCCATTTGTCTGT-3′ | a | |
| MIA | MIA3 | 5′-TTGCTGTCTGCCTTCTCCG-3′ | s | 355 bp |
| | MIA4 | 5′-GGCAGTAGAAATCCCATTTGTC-3′ | a | |

[a]From published sequences; [b]s = sense primer, as = antisense primer.

for the detection of melanoma cells in peripheral blood than the widely used tyrosinase PCR (La Valle et al, 1998). We investigated the presence of MIA mRNA in 19 human melanoma cell lines, 16 non-melanoma cell lines, 14 blood samples from melanoma patients, in six blood samples from non-melanoma cancer patients and in seven blood samples from healthy volunteers.

### MATERIALS AND METHODS

### Cell lines

Nineteen human melanoma cell lines and 16 non-melanoma cell lines were used in this study, most of these cell lines were described previously (De Vries et al, 1997). The melanoma cell lines were: BLM, MV3, MV1, MEL57, M14, 1F6, 1F6-m, 530, SK-MEL-28, A375P, A375M, Omel2, Bowes, MD3A, M24met, E10, 518A2, 603 and MZ2-MEL-3.0. The 16 non-melanoma cell lines were mainly tumour cell lines, all of them of various histological origin. These were: UMSCC2 (head-and-neck squamous cell carcinoma), CaCo2 (colorectal carcinoma), HEL (erythroleukemia), LB23 (sarcoma), LB37 (non-squamous cell lung carcinoma), HaCaT (immortalized keratinocytes), BB49 (head-and-neck cancer), A431 (cervical carcinoma), HFF (human foreskin fibroblasts), Jurkat (T-cell leukaemia), U937 (histiocytic lymphoma), Daudi (Burkitt's lymphoma), HT29 (colon adenocarcinoma), K562 (chronic myelogeneous leukaemia), HT1080 (fibrosarcoma) and MOLT-4 (acute lymphoblastic leukaemia). Suppliers of cell lines are mentioned elsewhere (De Vries et al, 1997), all other cell lines were from the American Type Culture Collection (Rockville, MD, USA). Cultured foreskin-derived melanocytes were also analysed.

### Blood samples

Blood samples were collected from seven healthy volunteers, six cancer patients without melanoma (two adenocarcarcinoma of the kidney, one adenocarcinoma of the pancreas, one small cell lung carcinoma, one testis carcinoma, one head-and-neck squamous cell carcinoma), seven melanoma patients who were previously tested negative for tyrosinase and MART-1 and seven melanoma patients who were previously tested positive for tyrosinase and MART-1 (De Vries et al, 1999).

### RNA isolation

To avoid contamination, separate rooms were used for RNA isolation, PCR, PCR analysis and sequencing. A one-way route and different laboratory clothing for each room was used, thus PCR product could not enter the RNA isolation laboratory. RNA isolation procedure from blood and from cell lines was described previously (De Vries et al, 1997, 1999).

### Reverse transcription

Reverse transcription was performed as described previously (De Vries et al, 1997, 1999). Briefly, 2 μg of total RNA was used for reverse transcription in 20 μl and supplied with 80 μl distilled water. From this mixture, 5 μl was used for each PCR, equal to 100 ng reverse transcribed RNA, hereby avoiding inaccurate pipetting of small volumes.

### PCR

The primers used in this study are listed in Table 1. Specific primers for phorbobilinogen deaminase (PBGD) and primers designated MIA1 and MIA2 were designed with the Primer Express software, version 1.0 (Perkin Elmer Applied Biosystems, Foster City, CA, USA). When designing these primers, melting temperatures of the primers were chosen between 58 and 60°C. MIA3 and MIA4 were suggested by others (La Valle et al, 1998). Both MIA primer combinations used in the PCR span the large 1 kb intron, both antisense primers MIA2 and MIA4 have sequences on both exon 3 and exon 4 (Figure 1).

PCR was performed as described (De Vries et al, 1997, 1999), optimal magnesium chloride concentration at 1.5 PBGD was amplified for 35 cycles of 0.5 min 94°C followed by 1.5 min at 60°C, MIA was amplified either with primer pairs MIA1 and MIA2 or with primer pairs MIA3 and MIA4, for 30 cycles and for 60 cycles at the same temperature and time scheme as PBGD. After each PCR, a final extension was performed for 3 min at 72°C.

### Sequence analysis

All three major PCR-bands were cut out of low-melting agarose and purified using Magic™ DNA clean-up kit (Promega, Madison, WI, USA) following the manufacturer's instructions. Subsequently, the PCR products were sequenced both in the 3′ and in the 5′ direction of the PCR product. During the labelling reaction, fluorescent dNTPs were incorporated, using the DNA sequencing kit (Perkin-Elmer Applied Biosystems, Warrington, UK). Fluorescent sequencing products were separated on a 6% polyacrylamide gel and analysed on an ABI PRISM 373 (Perkin-Elmer Applied Biosystems, Warrington, UK). Homology of the

*© 1999 Cancer Research Campaign*

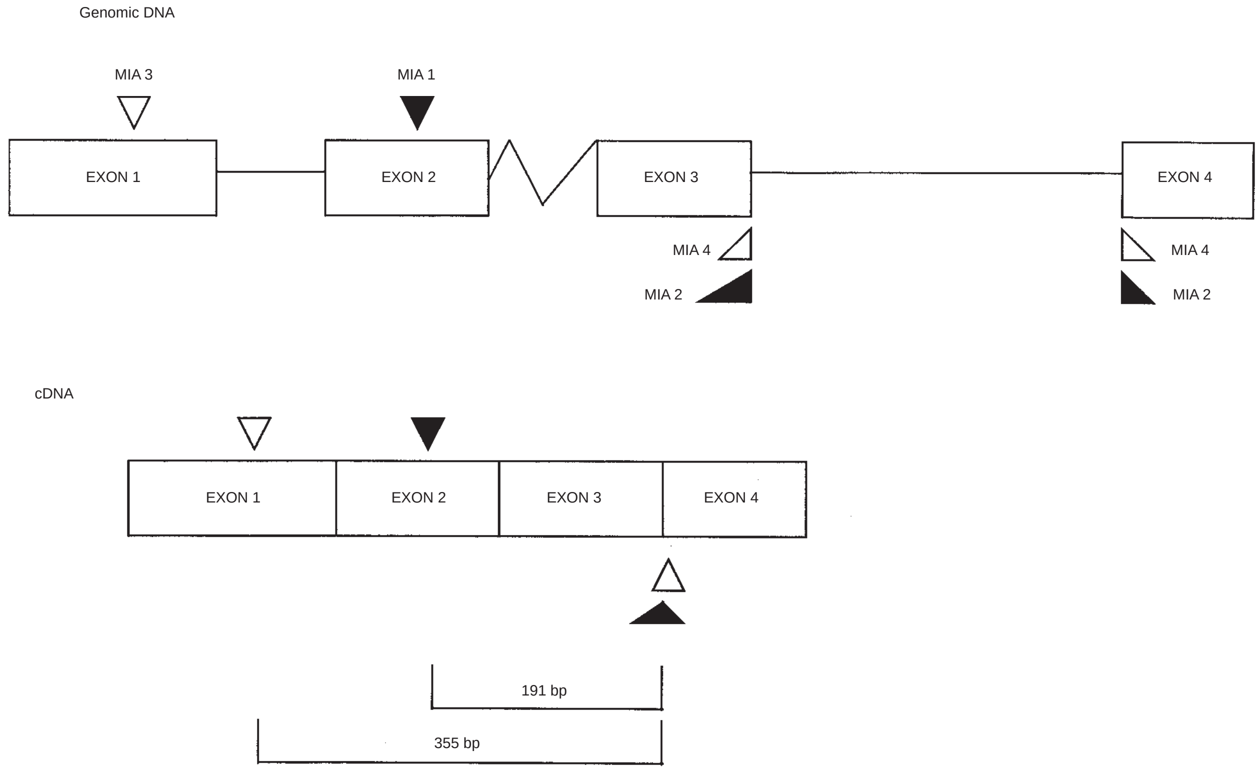

**Figure 1** Schematic representation of the primer pairs used for RT-PCR to detect MIA mRNA. Solid boxes represent exons. MIA2 and MIA4 are intron spanning primers

**Table 2** Presence of MIA transcripts as detected by 30 and 60 cycles of PCR with two MIA-specific primer sets

| Samples | MIA1,2 30 cycles | MIA1,2 60 cycles | MIA3,4 30 cycles | MIA3,4 60 cycles |
|---|---|---|---|---|
| Cell cultures and cell lines | | | | |
| Melanocytes | 1/1 | 1/1 | 1/1 | 1/1 |
| Melanoma cell lines | 16/19 | 19/19 | 16/19 | 19/19 |
| Non-melanoma cell lines | 7/16 | 16/16 | 7/16 | 16/16 |
| Blood samples | | | | |
| Blood healthy volunteers | 0/7 | 7/7 | 0/7 | 6/7 |
| Blood non-melanoma cancer patients | 0/6 | 5/6 | 0/6 | 5/6 |
| Blood melanoma patients, tyrosinase and MART-1 – | 0/7 | 7/7 | 0/7 | 7/7 |
| Blood melanoma patients, tyrosinase and MART-1 + | 0/7 | 7/7 | 0/7 | 6/7 |

sequences was analysed using pairwise alignment software (internetpage: http://dot.imgen.bcm.tcm.edu:9331/).

### RESULTS

### MIA mRNA in melanoma and non-melanoma cell lines

We first investigated the presence of MIA mRNA in melanoma and non-melanoma cell lines with 30 and 60 cycles of RT-PCR, independently with primer set MIA1 and MIA2 and with primer set MIA3 and MIA4. The results of a selection of these cell lines is displayed in Figure 2, the overall results are shown in Table 2. No genomic DNA was amplified with the primers used (results not shown).

From Figure 2 it is clear that the two sets of primers confirm each other. The majority of melanoma cell lines showed strong amplification of MIA after 30 cycles, with the exception of BLM, MV3 and E10. As reported previously (Bosserhoff et al, 1996), moderate amplification of MIA mRNA was observed in cultured melanocytes after 30 cycles of PCR. In the non-melanoma cell lines, strong MIA PCR signals after 30 cycles of amplification were observed for only two cell lines: HT29 and HT1080 (Figure 2). A slight MIA band was observed on ethidium bromide-stained gels in an additional five out of 16 non-melanoma cell lines (Jurkat, Daudi, K562, MOLT-4, U937) after 30 cycles of amplification. MIA was detected in all cell lines after 60 cycles of PCR amplification (Figure 2 and Table 2).

### MIA mRNA in blood of melanoma patients and of non-melanoma patients

Since it was reasoned that MIA could be a specific marker to detect melanoma cells in the peripheral blood with higher sensitivity and specificity than the thus far used tyrosinase PCR (La Valle et al, 1998), we performed 30 and 60 cycles of MIA PCR on blood samples of melanoma patients from a related study (De Vries et al, 1999).

We selected seven cDNA samples from melanoma patients who were previously consistently positive for MART-1 and compared the result with cDNA from seven blood samples from melanoma

 *© 1999 Cancer Research Campaign*

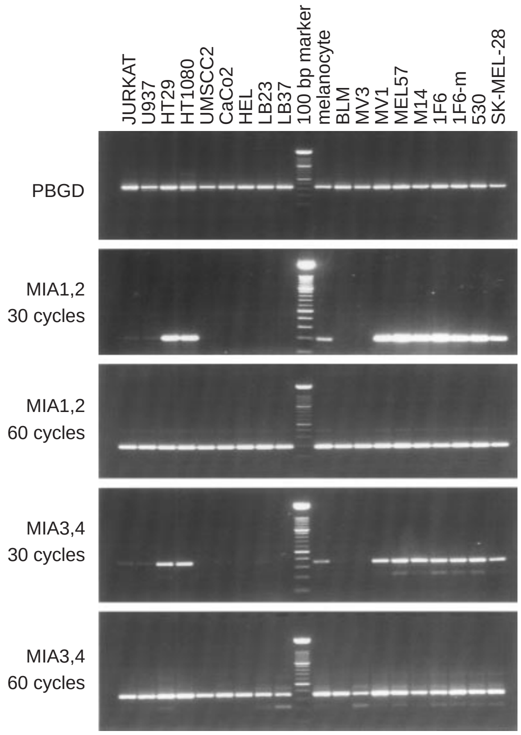

**Figure 2** Amplification of MIA in melanoma and in non-melanoma cell lines. cDNA of non-melanoma cell lines (to the left of the 100 bp marker) and melanocyte culture and melanoma cell lines (to the right of the 100 bp marker) was amplified with two MIA primer sets. As a test for amplifiability, the low copy housekeeping gene PBGD was amplified (upper panel) and MIA was detected with primer pairs MIA1 and MIA2 for 30 cycles (second panel) and 60 cycles (third panel) and with primer pairs MIA3 and MIA4 also for 30 cycles (fourth panel) and 60 cycles (fifth panel). Note that MIA was detected in most of the melanoma cell lines and in a few non-melanoma cell lines after 30 cycles and in all cell lines after 60 cycles of PCR

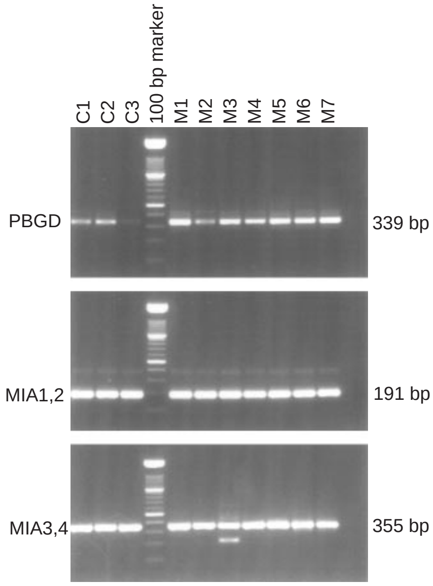

**Figure 3** Amplification of MIA in blood of healthy volunteers and melanoma patients. cDNA from blood of three healthy donors (C1–C3) and melanoma patients (M1–M7) was amplified with two MIA primer sets. M1–M4 were positive for melanoma marker MART-1, M5–M7 were negative for MART-1. As a test for amplifiability, the low copy housekeeping gene PBGD was amplified (upper panel) and MIA was visualized after 60 cycles of PCR with primer pairs MIA1 and MIA2 (second panel) and with primer pairs MIA3 and MIA4 (third panel). After 60 cycles of PCR, MIA was detectable in all blood samples analysed patients who were consistently negative for both tyrosinase and MART-1. Thirteen negative control blood samples, six from non-melanoma tumour-bearing patients with no history of primary melanoma and seven from healthy volunteers, were also tested for MIA mRNA. MIA mRNA was not detected in any blood sample after 30 cycles of PCR (not shown), nor in the melanoma samples, or in any of the control blood samples (Table 2). After 60 cycles of PCR, however, MIA was amplified in almost all blood samples (Figure 3, Table 2).

When the MIA primers were used in nested fashion (first PCR: MIA3 and MIA4, nested PCR: MIA1 and MIA2), the same results were obtained as with 60 cycles MIA3, MIA4 or MIA1, MIA2: MIA mRNA could be detected in all non-melanoma cell lines and peripheral blood samples tested (results not shown).

### Sequence analysis

The PCR products of 191 and 355 basepairs obtained by using primer pairs MIA1, MIA2 and MIA3, MIA4 respectively were analysed by sequence analysis. These PCR products were identical to the expected MIA cDNA sequence. Occasionally, an extra band of approximately 170 bp was observed with the primer pair MIA3 and MIA4, for example in Figure 3, lane M3. The subsequent sequence was identical to the 3′ part of the PCR product obtained by MIA3 and MIA4. Upon close analysis, the MIA3 primer showed high homology (82.4%, with only three mismatches and 100% identity in seven bases at the most 3′ region, neccesary for extention) with an internal MIA sequence, thus explaining the additional PCR-product.

### DISCUSSION

In the present study, we investigated whether a MIA-specific PCR can be added to the list of melanocyte-lineage-specific markers like tyrosinase and MART-1. To the best of our knowledge, tyrosinase and MART-1 are the only reliable markers for the detection of melanoma cells in blood (Hoon et al, 1995; Curry et al, 1998;; De Vries et al, 1999). Apart from melanocyte-lineage markers, specific tumour cell detection in peripheral blood with a PCR for the tumour-specific marker MAGE-3 has been reported for melanoma (Hoon et al, 1995) and in peripheral blood from patients with malignant tumours of diverse histology (Mori et al, 1997).

Although MIA expression was reported to be specific for melanoma cells and cells of neuroectodermal origin (Blesch et al, 1994; Bosserhoff et al, 1996), a large panel of melanoma versus non-melanoma cell lines was never compared. In our panel of 19 melanoma cell lines, we show that 16 out of 19 cell lines were positive for MIA after 30 cycles of PCR. Besides this, two out of 16 non-melanoma cell lines were positive at a similar level as the melanoma cell lines, and another five out of 16 cell lines showed faint expression of MIA. Discrepancies with an earlier report, where eight out of eight human non-melanoma cell lines did not show presence of MIA mRNA after 25 cycles of PCR (Bosserhoff et al, 1996), could be due to the additional five cycles used in our study and the difference in choice of non-melanoma cell lines. We

*© 1999 Cancer Research Campaign*

confirmed our results with two sets of MIA primers. Though we found MIA mRNA in melanocytes, a previous study denies this presence in melanocytes (Bosserhoff et al, 1996). An explanation may be that the melanocytes used in our study were cultured in the presence of PMA, which has been described as a stimulator for MIA expression (Bosserhoff et al, 1996). It was reported that MIA expression inversely correlates with pigmentation in melanoma metastases (Van Groningen et al, 1995). We previously described the expression of melanocyte differentiation genes gp100, tyrosinase and MART-1 in the same panel of cell lines as used in this study (De Vries et al, 1997). We cannot confirm the apparent inverse correlation in lesions with cell lines: the MIA-negative melanoma cell lines BLM, MV3 and E10 did not express any of the pigmentation genes. Other obvious correlations with pigmentation genes and MIA expression fail to attract our attention: melanoma cell lines Bowes, MD3A and M24met express MIA but none of the pigmentation genes (De Vries et al, 1997). Promising results were obtained with immunochemical determinations of MIA in serum or plasma from melanoma patients, where the detection of MIA was more sensitive than detection of S100 protein (Bosserhoff et al, 1997). At the mRNA level, however, our study clearly shows that MIA mRNA can be detected in any cell type, including peripheral blood cells when using enough PCR cycles. The MIA gene can be listed as one of many 'illegitimately transcribed' genes, where illegitimate transcription is defined as transcription of a gene in any cell type (Chelly et al, 1989), which was previously reported for melanoma antigens gp100 (Keilholz, 1996; Brouwenstijn et al, 1997; Curry et al, 1998), MUC18 (Curry et al, 1998) and p97 (Curry et al, 1998). Thus, our results do not prove that a MIA RT-PCR is of value in the detection of minute tumour load of melanoma cells in the peripheral blood or in the target organs of the metastatic process.

### ACKNOWLEDGEMENTS

Ine Cornelissen and Han Zendman are acknowledged for providing cDNA from melanocytes and some non-melanoma cell lines, Louis van de Locht from the Central Hematology Laboratory is acknowleged for the design of MIA-primers. Elly Petri and co-workers from the Bloodbank Gelderse Rivieren has provided us generously with blood samples. This work was supported by a grant number NUKC 95-912 from the Dutch Cancer Society.

### REFERENCES

Blesch A, Bosserhoff A-K, Apfel R, Behl C, Hessdoerfer B, Schmitt A, Jachimczak P, Lottspeich F, Buettner R and Bogdahn U (1994) Cloning of a novel malignant melanoma-derived growth-regulatory protein, MIA. *Cancer Res* **54**: 5695–5701

Bosserhoff A-K, Hein R, Bogdahn U and Buettner R (1996) Structure and promoter analysis of the gene encoding the human melanoma-inhibiting protein MIA. *J Biol Chem* **271**: 490–495

Bosserhoff A-K, Kaufmann M, Kaluza B, Bartke I, Zirngibl H, Hein R, Stolz W and Buettner R (1997) Melanoma-inhibiting activity, a novel serum marker for progression of malignant melanoma. *Cancer Res* **57**: 3149–3153

Brossart P, Keilholz U, Willhauck M, Scheibenbogen C, Mohler T and Hunstein W (1993) Hematogenous spread of malignant melanoma cells in different stages of disease. *J Invest Dermatol* **101**: 887–889

Brossart P, Schmier J-W, Kruger S, Willhauck M, Scheibenbogen C, Mohler T and Keilholz U (1995) A polymerase chain reaction-based semiquantitative assessment of malignant melanoma cells in peripheral blood. *Cancer Res* **55**: 4065–4068

Brouwenstijn N, Slager EH, Bakker ABH, Schreurs MWJ, Van der Spek CW, Adema GJ, Schrier PI and Figdor CG (1997) Transcription of the gene encoding melanoma-associated antigen gp100 in tissues and cell lines other than those of the melanocytic lineage. *Br J Cancer* **76**: 1562–1566

Burchill SA, Bradbury FM, Pittman K, Southgate J, Smith B and Selby P (1995) Detection of epithelial cancer cells in peripheral blood by reverse transcription-polymerase chain reaction. *Br J Cancer* **71**: 278–281

Chelly J, Concordet J, Kaplan J and Kahn A (1989) Illegitimate transcription: transcription of any gene in any cell type. *Proc Natl Acad Sci USA* **86**: 2617–2621

Curry BJ, Myers K and Hersey P (1998) Polymerase chain reaction detection of melanoma cells in the circulation: relation to clinical stage, surgical treatment, and recurrence from melanoma. *J Clin Oncol* **16**: 1760–1769

De Vries TJ, Fourkour A, Wobbes T, Verkroost G, Ruiter DJ and Van Muijen GNP (1997) Heterogeneous expression of immunotherapy candidate proteins gp100, MART-1 and tyrosinase in human melanoma cell lines and in human melanocytic lesions. *Cancer Res* **57**: 3223–3229

De Vries TJ, Fourkour A, Punt CJA, Van de Locht LT, Wobbes T, Van den Bosch S, De Rooij MJM, Mensink EJ, Ruiter DJ and Van Muijen GNP (1999) Reproducibility of detection of tyrosinase and MART-1 transcripts in the peripheral blood of melanoma patients: a quality control study using real-time quantitative PCR. *Br J Cancer* **80**: 883–891

Hoon DS, Wang Y, Dale PS, Conrad AJ, Schmid P, Garrison D, Kuo C, Foshag LJ, Nizze AJ and Morton DL (1995) Detection of occult melanoma cells in the blood with a multiple-marker polymerase chain reaction assay. *J Clin Oncol* **13**: 2109–2116

Keilholz U (1996) Diagnostic PCR in melanoma: methods and quality assurance. *Eur J Cancer* **32**: 1661–1663

Keiholz U, Willhauck M, Scheibenbogen C, De Vries TJ and Burchill S (1997) Polymerase chain reaction detection of circulating tumour cells. *Melanoma Res* **7**: S133–S141

Kunter U, Buer J, Probst M, Duensing S, Dallmann I, Grosse J, Kirchner H, Schluepen EM, Volkenandt M, Ganser A and Atzpodien J (1996) Peripheral blood tyrosinase messenger RNA detection and survival in malignant melanoma. *J Natl Cancer Inst* **88**: 590–594

La Valle GJ, Williams NY, Walker MJ, Triozzi PL and Barbera-Guillem E (1998) Expression of melanoma inhibitory activity in the peripheral blood of melanoma patients by the reverse transcription-polymerase chain reaction (RT-PCR). *Proc Am Assoc Cancer Res* **39**: 229(Abstract)

Mellado B, Colomer D, Castel T, Munoz M, Carballo E, Galan M, Mascaro JM, Vives-Corrons JL, Grau JJ and Estape J (1996) Detection of circulating neoplastic cells by reverse-transcriptase polymerase chain reaction in malignant melanoma: association with clinical stage and prognosis. *J Clin Oncol* **14**: 2091–2097

Mori M, Mimori K, Tanaka F, Ueo H, Sugimachi K and Akiyoshi T (1997) Molecular diagnosis of circulating cancer cells using MAGE gene assays. *JAMA* **278**: 476–477

Smith B, Selby P, Southgate J, Pittman K, Bradley C and Blair GE (1991) Detection of melanoma cells in peripheral blood by means of reverse transcriptase and polymerase chain reaction. *Lancet* **338**: 1227–1229

Van Groningen JJM, Bloemers HPJ and Swart GWM (1995) Identification of melanoma inhibitory activity and other differentially expressed messenger RNAs in human melanoma cell lines with different metastatic capacity by messenger RNA differential display. *Cancer Res* **55**: 6237–6243

Wimmer M, Meyer JC, Seifert B, Dummer R, Flace A and Burg G (1997) Prognostic value of serum 5-S-cysteinyldopa for monitoring human metastatic melanoma during immunochemotherapy. *Cancer Res* **57**: 5073–5076

 *© 1999 Cancer Research Campaign*
